# Calibrating the Extended Hückel Method to Quantitatively Screen the Electronic Properties of Materials

**DOI:** 10.1038/s41598-018-28864-2

**Published:** 2018-07-12

**Authors:** Linda P. Grabill, Robert F. Berger

**Affiliations:** Department of Chemistry, Western Washington University, Bellingham, WA USA

## Abstract

The extended Hückel (eH) tight-binding method has historically been prized for its computational ease and intuitive chemical clarity. However, its lack of quantitative predictiveness has prevented the eH method from being used as a tool for rapidly screening materials for desired electronic properties. In this work, we demonstrate that when eH input parameters are calibrated using density functional theory (DFT) calculations of carefully chosen sets of simple crystals, the eH parameters retain most of their quantitative accuracy when transferred to more complex, structurally related phases. Using solar-energy-relevant semiconductors and insulators in the Sr–Ti–O family as a case study, we show that calibrated eH parameters can match the features of DFT band structures within about two tenths of an eV, at a tiny fraction of the computational cost of DFT.

## Introduction

Density functional theory (DFT)-based methods^1,2^ are generally seen as the state of the art in quantitatively computing the electronic structure of materials. DFT has demonstrated the ability to match experiment closely enough to be valuable in screening and predicting the electronic properties of existing and new materials. With improvements in both hardware and methodology, the efficiency of DFT has reached the point that it is frequently used in high-throughput screening studies of bulk solids and highly-ordered surfaces^3–7^. However, the structures of interest in modern materials science are not always simple enough to be tractable in high-throughput DFT studies. They may be doped, layered, or nanostructured materials, for which accurate calculations require hundreds or thousands of atoms per unit cell. Even with advances in hardware and software, there will thus remain a need for more efficient techniques that can push the limits of complexity, quickly computing the properties of large numbers of structures with hundreds or thousands of atoms. Such methods are valuable as rough screening tools even if their results are more approximate than those of DFT.

In this work, we explore and assess the extent to which the extended Hückel (eH) tight-binding method can be employed as a screening tool for the electronic properties (e.g., band gaps and densities of states) of complex solid structures. The eH method, developed by Hoffmann in 1963^8^, is prized for its ability to transparently highlight chemical trends in solids^9^. While the eH method is generally limited in its quantitative accuracy, it has been shown in several studies^10–17^ that when eH input parameters are properly calibrated, an eH-computed electronic band structure can quantitatively resemble its DFT-computed analog. This past work in eH parameter calibration has primarily aimed to develop intuitive, orbital-based chemical understanding. Even when quantitative transferability has been a goal^10^, the predictiveness of eH calculations has been limited by the fact that parameter values are calculation-specific–that is, a separate calibration to DFT must be performed for each chemical structure.

In contrast, our goal is to test the *a priori* predictiveness of calibrated eH parameters. This paper is a demonstration that when parameters are calibrated using carefully chosen sets of simpler compounds, the eH method can quantitatively capture the electronic structure of complex materials without the need for much more expensive DFT calculations. As a test set, we focus on prototypical semiconductors, insulators, and their tunable variants in the Sr–Ti–O family, some of which are of interest in solar energy conversion applications. This work is intended to provide a road map for, and identify the limitations of, the broader goal of using the eH method to mine the universe of doped, layered, and otherwise modified materials for desired electronic properties. By calibrating eH band structures to DFT results, we ensure that our approach can evolve and improve along with DFT methodology, as our calibration process can be adapted to new density functionals on higher rungs of the so-called Jacob’s ladder^18^ at no additional computational cost.

## Methods and Approach to Parameter Optimization

**Figure 1:**
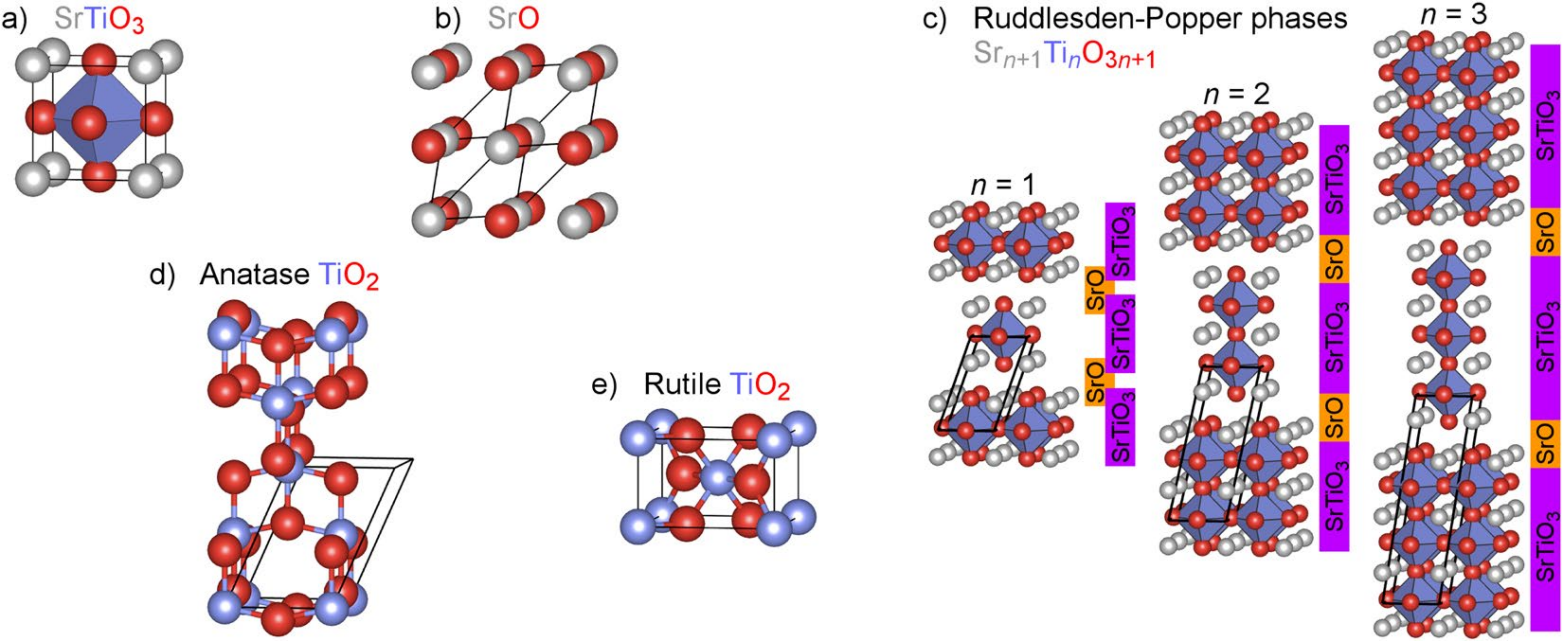
Crystal structures computed in this paper: (**a**) cubic perovskite SrTiO_3_, (**b**) NaCl-type SrO, (**c**) the *n* = 1−3 members of the Sr–Ti–O Ruddlesden-Popper series, (**d**) anatase TiO_2_, and (**e**) rutile TiO_2_. In all cases, the unit cell calculated is the primitive unit cell highlighted in black.

All DFT geometry optimizations and electronic structure calculations are performed using the VASP package^19^ and PAW potentials^20^. To demonstrate the ability of calibrated eH calculations to match DFT results regardless of functional, our DFT calculations use three fundamentally different functionals: the local-density approximation (LDA), the Perdew-Burke-Ernzerhof (PBE) generalized-gradient approximation^21^, and the Heyd-Scuseria-Ernzerhof (HSE06) hybrid functional^22^. Of these three functionals, LDA and PBE are known to underestimate band gaps relative to experiment–a feature that will be imparted to eH when those functionals are used for calibration. It is important to be aware of the fact that the eH method can only aim to match experimental electronic properties as well as the DFT functional to which it is calibrated. Electrons taken to be valence in these DFT calculations are Sr 4*s*^2^4*p*^6^5*s*^2^, Ti 3*s*^2^3*p*^6^4*s*^2^3*d*^2^, and O 2*s*^2^2*p*^4^. A 6 × 6 × 6 *k*-point mesh is used for the unit cell of cubic perovskite SrTiO_3_ (Fig. 1a), and numbers of *k*-points are scaled inversely with unit cell size to yield similar precision for all compounds. All DFT-computed crystal structures in this work are provided in the Supplementary Information.

Semi-empirical eH electronic structure calculations are performed using the YAeHMOP package^23^. Crystal structures in these calculations are optimized using the DFT functional to which each eH calculation is calibrated. Though the future utility of our parameter calibration process as an approach to materials screening will require that structures are generated without the need for DFT geometry optimization, the use of DFT geometries in this work allows for the clearest comparison between DFT and eH.

More detailed coverage of the eH methodology and its Slater-type orbitals and associated input parameters are provided in the Supplementary Information, and in the original work of Hoffmann^8^. To summarize these issues, each element’s valence *s* orbital requires two input parameters: *H*_*ii*,*s*_ (which roughly corresponds to the atomic orbital’s energy) and ${\zeta }_{s}$ (which governs the spatial extent of the basis function). Each element’s set of valence *p* orbitals requires an additional *H*_*ii*,*p*_ and *ζ*_*p*_. When applicable, an element’s set of valence *d* orbitals requires four parameters: *H*_*ii*,*d*_, *ζ*_1_, *ζ*_2_, and the ratio of coefficients of the two exponential functions *c*_2_/*c*_1_. Finally, the calculation as a whole requires a Wolfsberg-Helmholtz constant, *K*, traditionally taken to be 1.75. For greater flexibility in our calibrations, we allow *K* to vary as an adjustable parameter (which, as shown in the Supplementary Information, typically converges to a value of approximately 3). We find that allowing *K* to vary speeds up our calibration process significantly, and increases the likelihood of an eH band structure converging to match DFT results. The input parameters in an eH calculation are summarized in Table 1.

**Table 1:** The number and identity of input parameters in an eH calculation. More information about the physical interpretation of these parameters is given in the Supplementary Information.

	Number of parameters	Identity of parameters
Element with valence *s* and *p* orbitals	4	*H*_*ii*,*s*_, ζ_*s*_, *H*_*ii*,*p*_, ζ_*p*_
Element with valence *s*, *p*, and *d* orbitals	8	*H*_*ii*,*s*_, ζ_*s*_, *H*_*ii*,*p*_, ζ_*p*_, *H*_*ii*,*d*_, ζ_1_, *ζ*_2_, *c*_2_/*c*_1_
Additional parameter (Wolfsberg-Helmholtz constant)	1	*K*

Our approach to calibrating eH input parameters to DFT calculations draws inspiration from the groups of Cerdá^10^ and Fredrickson^17,24^. For a chemically meaningful set of valence and conduction bands of interest near the Fermi energy, the root-mean-squared deviation (RMSD) is computed between DFT and eH energies over a uniform grid of *k*-points:

$${\rm{RMSD}}=\sqrt{\sum _{j}^{{N}_{{\rm{energies}}}}\frac{{({E}_{j}^{{\rm{eH}}}-{E}_{j}^{{\rm{DFT}}})}^{2}}{{N}_{{\rm{energies}}}}}$$

At each *k*-point, the bands are compared in order of their energies (i.e., the lowest-energy eH and DFT bands within the calibrated range are compared, the second-lowest-energy bands are compared, etc.). Traditionally, the input parameters representing atomic orbital energies in eH calculations (*H*_*ii*_) are taken to be ionization energies, implicitly defining the vacuum level to be zero energy. To ensure fair comparison to DFT, we therefore adjust DFT orbital energies by the approximate correction term given in each DFT calculation (defined in VASP as “alpha + bet”), effectively setting the vacuum energy to zero. We find that anchoring the band structures to an unambiguous definition of zero energy (rather than comparing band energies only in a relative sense) is necessary in order for eH parameters to converge to physically reasonable values. Unlike some previous work^17^, our parameter calibration does not rely on comparing the two methods’ projected densities of states. This simplification is sensible because our focus is on semiconductors for which bands of different atomic orbital character are well separated in energy.

We calibrate sets of eH input parameters to minimize RMSDs between eH and DFT calculations. When eH parameters are calibrated based on sets of multiple compounds, it is the root-mean-squared value of the RMSDs of each compound that is minimized. The simultaneous calibration of eH parameters requires the minimization of a many-dimensional function. In such problems, there does not exist an optimization algorithm that guarantees the discovery of a globally optimal parameter set. We employ the Nelder-Mead simplex method^25^, restarting the algorithm every hundred steps in order to more effectively explore broad regions of parameter space. While our work will illustrate that YAeHMOP’s default eH input parameters lead to quantitatively poor matches to DFT band structures, default parameters (which order the energies of atomic orbitals correctly) are typically good starting points for calibration. After experimenting with sets of randomly-generated input parameters, we have found that the best calibrations start from default parameters or from calibrated parameters for other compounds or functionals. Neither our approach nor any other can ensure globally optimal parameter sets. It is likely that values of the eH parameters are correlated, and a variety of parameter sets would match DFT band structures approximately equally well. The success of our work to develop sets of quantitatively useful, physically reasonable eH parameters is best assessed by their transferability to and predictiveness of related compounds.

## Results and Discussion

### Compounds of Interest

We intend to explore how well eH parameters calibrated for a set of structurally simple compounds can quantitatively capture the electronic band structures of more complex, related compounds. As a case study, we choose to investigate semiconductors and insulators in the Sr–Ti–O family, illustrated in Fig. 1. We begin with SrTiO_3_, which adopts the centrosymmetric cubic perovskite structure (Fig. 1a) at room temperature and has an indirect optical band gap of approximately 3.2 eV^26,27^. Due to the promise that SrTiO_3_ has shown in solar water-splitting applications for several decades^28–31^ and the widespread interest in tuning its electronic structure^32–37^, it is crucial that computational methods are able to accurately capture the band gap and band-edge orbital energies and character of SrTiO_3_. As the other “parent” compound in our calibration set, we choose SrO, an insulator which adopts the NaCl structure (Fig. 1b).

After calibrating the eH parameters of SrTiO_3_ and SrO, we test the performance of these calibrated parameters for compounds outside the calibration set. We investigate the *n* = 1−3 members of the Ruddlesden-Popper series^38,39^ (Sr_*n*+1_ Ti_*n*_ O_3*n*+1_, Fig. 1c), which have several appealing features for this study. First, they are structurally related to SrTiO_3_ and SrO, in that they have regions that locally resemble the SrTiO_3_ and SrO structures (Fig. 1c), and ionic charges of Sr ^2+^, Ti ^4+^, and O ^2−^. Second, the Ruddlesden-Popper phases can be seen as more complex (i.e., more expensive to compute) variants of SrTiO_3_ and SrO. As a test of our approach, it is therefore interesting to explore how well parameters calibrated using SrTiO_3_ and SrO transfer to these compounds. Third, because the Sr–Ti–O Ruddlesden-Popper phases have been investigated with an eye toward band gap engineering^40^, the ability to accurately compute their electronic band structure is important.

In the final section of this paper, we test the quality of our calibrated eH parameters in computing both the anatase (Fig. 1d) and rutile (Fig. 1e) phases of TiO_2_. TiO_2_ has similarities with SrTiO_3_ in both structure and applications. Both have Ti ^4+^ cations approximately octahedrally coordinated by six O ^2−^ anions. Like SrTiO_3_, TiO_2_ has generated interest in water-splitting photocatalysis for several decades^30,31,41,42^ (in part due to the 3.4^43^ and 3.0 eV^44,45^ band gaps of anatase and rutile), as well as efforts to tune its electronic structure^46–50^. TiO_2_ therefore provides another interesting test of how effectively calibrated eH parameters can be transferred to related compounds.

### Calibration of SrTiO_3_ Parameters

Before calibrating the eH input parameters of the cubic perovskite SrTiO_3_ (Fig. 1a), we must select which bands to include in the calibration process. In general, the chemical and physical properties of a material depend most strongly on the valence and conduction bands (i.e., the frontier orbitals). The choice of how many valence and conduction bands to include in calibration is a tradeoff in a sense. Adding additional bands far from the band gap provides more points of comparison, but can negatively affect the calibration of bands closer to the band gap. For the compounds we explore, in which blocks of bands are well separated based on their atomic orbital character, choices are relatively straightforward. In the case of SrTiO_3_, the valence bands consist primarily of filled O 2*p* states, while the conduction bands consist primarily of unfilled Ti 3*d* states–and more narrowly, of *t*_2*g*_ states, the three lowest-energy Ti 3*d* bands. In comparing DFT and eH band structures of SrTiO_3_, we therefore focus on the nine highest-energy filled bands (the 2*p* states of the three O atoms) and the three lowest-energy unfilled bands (the *t*_2*g*_ states of the Ti atom). Another decision we must make is whether to include Sr 4*d* orbitals in the eH basis set. As with all main-group elements, Sr *d* orbitals are often neglected in our chemical intuition and in the default basis sets of simple calculations. While Sr 4*d* orbitals are not the primary components of the valence or conduction bands of SrTiO_3_, they may play a secondary role. We therefore calibrate eH parameter sets both without and with Sr 4*d* orbitals.

**Figure 2:**
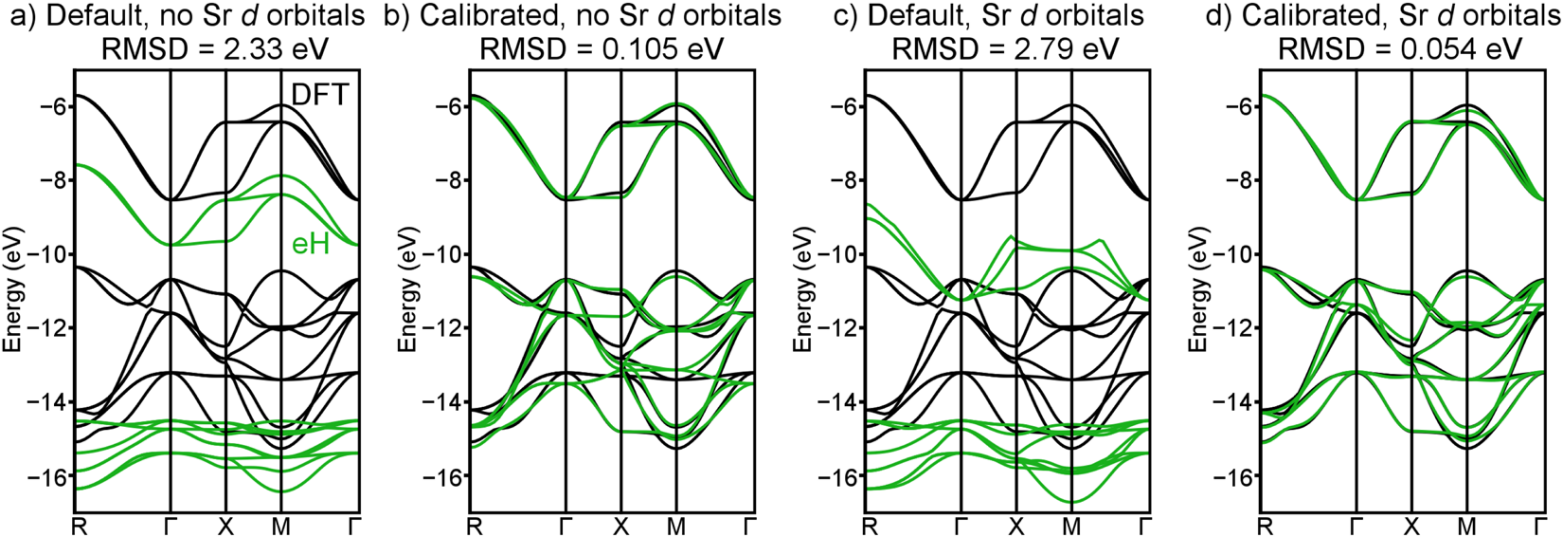
Comparisons of the DFT-LDA (black) and eH (green) band structures of SrTiO_3_ at various levels of eH parameter calibration: (**a**) default and (**b**) calibrated eH parameters without Sr 4*d* orbitals included, and (**c**) default and (**d**) calibrated eH parameters with Sr 4*d* orbitals included.

When Sr 4*d* orbitals are not included in the eH basis set, the default parameters given in the YAeHMOP code result in a quantitatively poor match to DFT-LDA (Fig. 2a). Though shapes and degeneracies in the eH valence and conduction bands show some similarity to DFT, their band widths and absolute energies differ by a margin (RMSD = 2.33 eV) large enough to prevent default eH parameters from having any real quantitative predictive value. When the eH parameters are calibrated, the situation improves dramatically (Fig. 2b). The shapes and absolute energies of DFT and eH bands match well enough (RMSD = 0.105 eV, a more than 20-fold improvement) to suggest that, when parameters are calibrated, eH calculations have the potential to be quantitatively predictive.

When Sr 4*d* orbitals are included in the eH basis set, the importance of parameter calibration becomes even clearer. Default parameters (Fig. 2c) yield band shapes in the eH band structure that differ qualitatively from DFT (RMSD = 2.79 eV). [Because no default 4*d* parameters are provided for Sr in the YAeHMOP package, we instead use the default 4*d* parameters of Zr, the nearest element in the periodic table for which 4*d* parameters are provided.] However, when calibrated (Fig. 2d), the match between DFT and eH is extremely close (RMSD = 0.054 eV, a more than 50-fold improvement). The quality of parameters calibrated to the PBE and HSE06 functionals is similar to LDA. This suggests that eH calculations can potentially achieve the accuracy of any density functional. The eH parameter sets for these calculations are given in the Supplementary Information.

### Simultaneous Calibration of SrTiO_3_ and SrO Parameters

Having explored the eH parameters for SrTiO_3_, we expand the scope of our calibration to include SrO. Because the elements in SrO are a subset of those in SrTiO_3_, SrO can in principle be computed using eH parameters calibrated for SrTiO_3_. However, parameters calibrated for one compound are not necessarily suitable when applied to another. By identifying parameters that are simultaneously suitable for both SrTiO_3_ and SrO, we are preparing for calculations of the more complicated Ruddlesden-Popper structures, which consist of local regions of the SrTiO_3_ and SrO structures (Fig. 1c). For SrTiO_3_, we included filled O 2*p* and unfilled Ti 3*d* states in our band structure comparisons. In order to be consistent, we therefore include only the three highest-energy filled bands of SrO (the O 2*p* states, as SrO contains no Ti atoms).

**Figure 3:**
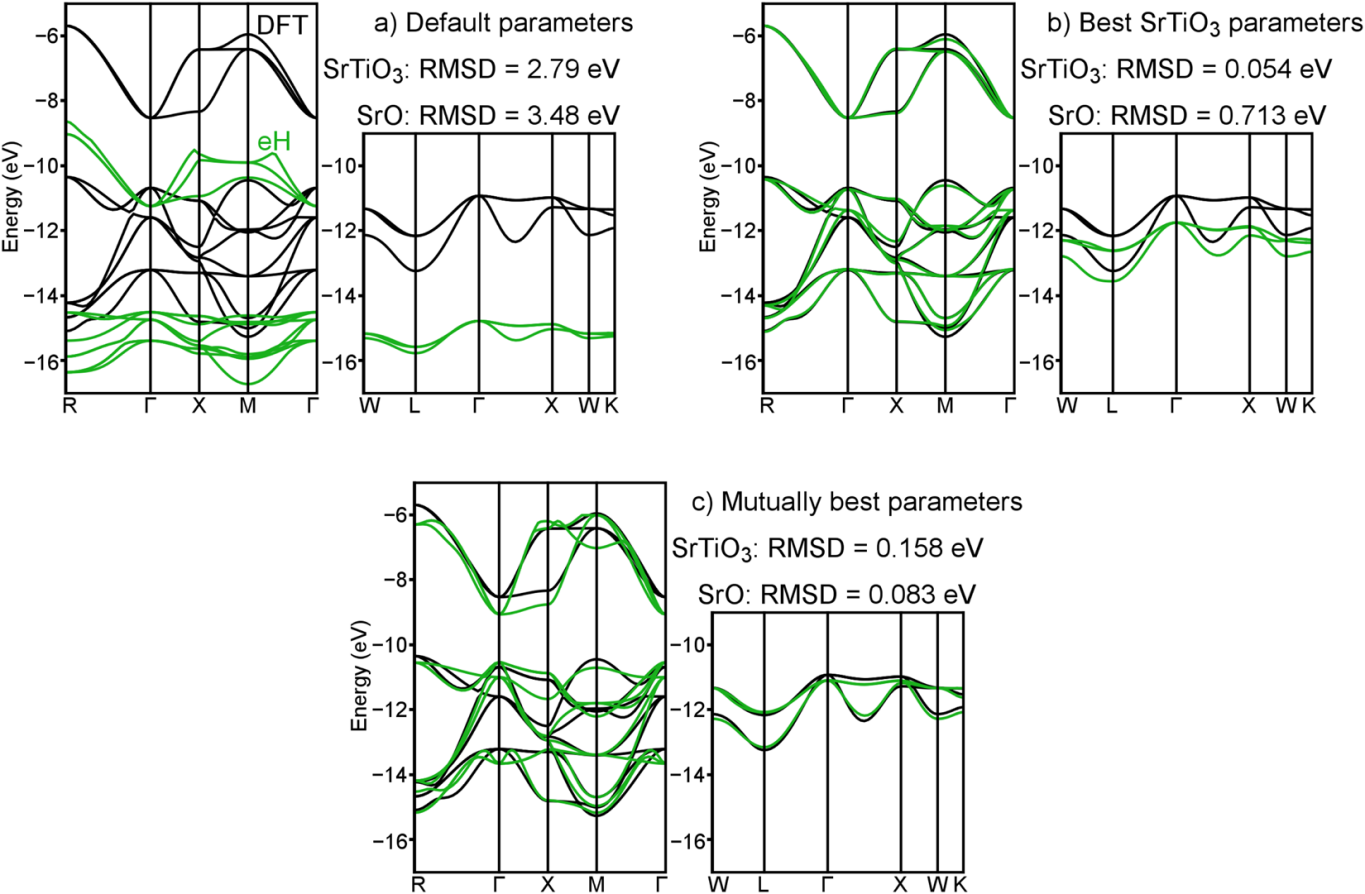
Comparisons of the DFT-LDA (black) and eH (green) band structures of SrTiO_3_ (left) and SrO (right) at various levels of simultaneous eH parameter calibration (with Sr 4*d* orbitals included): (**a**) default parameters, (**b**) parameters calibrated to provide the best match for SrTiO_3_, and (**c**) parameters calibrated to provide the best compromise between SrTiO_3_ and SrO.

In Fig. 3, band structures of SrTiO_3_ and SrO computed using the eH method (with Sr 4*d* orbitals included in the basis set) are compared to those computed using DFT-LDA. As was the case for SrTiO_3_, default eH parameters do a somewhat reasonable job capturing the qualitative DFT band shapes and degeneracies of SrO, but a poor job (RMSD = 3.48 eV) capturing the quantitative band widths and energies (Fig. 3a). When the parameters calibrated using SrTiO_3_ are applied to SrO (Fig. 3b), the band widths and absolute energies improve somewhat relative to default parameters, but still differ enough from DFT (RMSD = 0.713 eV) to limit the quantitative conclusions one could draw from an eH calculation. It is only when input parameters are simultaneously calibrated for SrTiO_3_ and SrO that eH calculations of both compounds come into reasonable quantitative agreement with DFT (Fig. 3c). Not surprisingly, the inclusion of SrO worsens the match between the DFT and eH band structures of SrTiO_3_, raising the RMSD from 0.054 eV to 0.158 eV. However, this comes with the benefit of improving the RMSD between the DFT and eH band structures of SrO from 0.713 eV to 0.083 eV, in effect reaching a compromise in which a single set of eH parameters can capture the electronic structures of both SrTiO_3_ and SrO to a degree that is potentially useful as a quantitative screening tool.

Table 2 summarizes the quality of eH parameters calibrated by simultaneously considering SrTiO_3_ and SrO. For both SrTiO_3_ alone and for simultaneous parameter calibration, eH parameters can be calibrated to similarly match LDA, PBE, and HSE06 results. One notable feature of Table 2 is that eH calculations of SrO cannot closely match DFT unless Sr 4*d* orbitals are included in the eH basis set. While Sr 4*d* orbitals are not the primary component of the valence bands, it is clear that their presence shapes the valence bands in significant ways. The eH parameter sets corresponding to the results in Table 2 are given in the Supplementary Information.

**Table 2:** Root-mean-squared deviations (RMSDs) of eH band structures of SrTiO_3_ and SrO from their DFT counterparts. Comparisons are made for default eH parameters, parameters calibrated to provide the best match for SrTiO_3_, and parameters calibrated to provide the best compromise between SrTiO_3_ and SrO.

Density functional	Type of eH parameters	RMSD for SrTiO_3_, without Sr *d* (eV)	RMSD for SrTiO_3_, with Sr *d* (eV)	RMSD for SrO, without Sr *d* (eV)	RMSD for SrO, with Sr *d* (eV)
LDA	Default	2.33	2.79	3.10	3.48
	SrTiO_3_	0.105	0.054	0.541	0.713
	SrTiO_3_/SrO	0.161	0.158	0.263	0.083
PBE	Default	2.92	3.35	3.86	4.20
	SrTiO_3_	0.084	0.045	0.623	0.788
	SrTiO_3_/SrO	0.158	0.159	0.216	0.092
HSE06	Default	2.05	2.65	2.57	2.92
	SrTiO_3_	0.099	0.049	0.732	0.911
	SrTiO_3_/SrO	0.190	0.193	0.240	0.109

### Transfer of Parameters to Related Compounds

Until this point, we have demonstrated that eH calculations can achieve quantitative accuracy for given compounds when the input parameters are calibrated using those same compounds. Of course, the need to perform a DFT calculation on every compound would defeat much of the appeal of the eH method as a screening tool. Ideally, we hope to use simple structures to calibrate sets of eH parameters that retain their quantitative accuracy when applied to much more complex structures. For the remainder of our work, we explore the extent to which calibrated eH parameters can be transferred to classes of similar compounds.

In this section, we make one methodological change that is worth noting. In our parameter calibrations, it was useful to compare the absolute DFT and eH band energies (with the vacuum level defined as zero energy) in order to ensure physically reasonable eH parameters. However, in testing whether our calibrated parameters can effectively be transferred, it is the *differences* between energies that determine many properties of interest (e.g., solar absorption efficiency). Henceforth, when computing RMSDs between DFT and eH band structures, we therefore allow a rigid shift of the DFT band energies that brings the DFT and eH band structures into their closest match.

**Figure 4:**
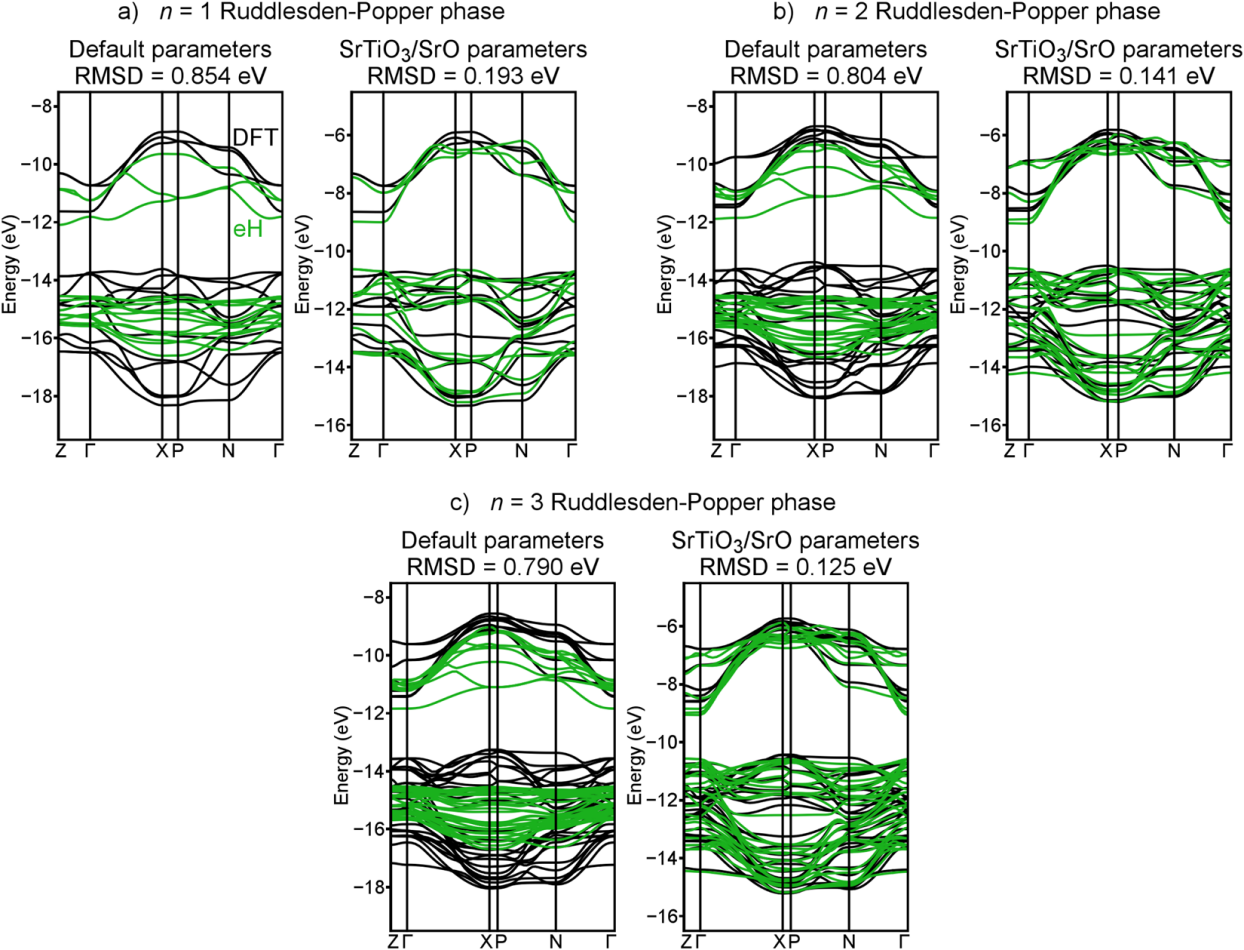
Comparisons of the DFT-LDA (black) and eH (green) band structures of the first three members of the Sr–Ti–O Ruddlesden-Popper series, Sr_*n*+1_ Ti_*n*_ O_3*n*+1_: (**a**) *n* = 1, (**b**) *n* = 2, and (**c**) *n* = 3. Default eH parameters (with Sr 4*d* orbitals included) are shown on the left, and parameters calibrated by simultaneously considering SrTiO_3_ and SrO are shown on the right.

In Fig. 4, band structures of the *n* = 1–3 Ruddlesden-Popper phases (Fig. 1c) computed using the eH method (with Sr 4*d* orbitals included in the basis set) are compared to those computed using DFT-LDA. Even with the DFT band structures rigidly shifted to ensure that DFT and eH are centered at the same energy, default eH parameters yield poor matches. With default parameters (Fig. 4, left), RMSDs are approximately 0.8 eV for the Ruddlesden-Popper phases, with visible qualitative differences between the two methods. With the parameters that were calibrated by simultaneously considering SrTiO_3_ and SrO (Fig. 4, right), RMSD’s fall below 0.2 eV, with visibly strong agreement between the band shapes and energies computed with the two methods. These calibrated eH parameters lead to more than 4-fold improvement over default parameters, and they match DFT to a quantitative degree that is potentially useful as a screening tool.

Table 3 summarizes the extent to which various eH parameters match DFT calculations of the *n* = 1–3 Ruddlesden-Popper phases using the LDA and PBE functionals. Once again, it appears that eH can be calibrated to similarly match different DFT functionals. When transferring calibrated parameters to the Ruddlesden-Popper phases, there is little if any benefit to including Sr 4*d* orbitals in the calibration process. Interestingly, parameters calibrated by simultaneously considering SrTiO_3_ and SrO perform better for phases with small *n*, while parameters calibrated using only SrTiO_3_ perform better for large *n*. This can be understood if one considers that in phases with larger *n*, a larger fraction of the structure resembles SrTiO_3_ and a smaller fraction resembles SrO. This suggests the importance of calibrating eH parameters using a set of compounds that closely resemble the complex structures one is interested in. The eH parameter sets corresponding to the results in Table 3 are given in the Supplementary Information.

**Table 3:** Root-mean-squared deviations (RMSDs) of eH band structures of the first three members of the Sr–Ti–O Ruddlesden-Popper series (Sr_*n*+1_ Ti_*n*_ O_3*n*+1_) from their DFT counterparts. Comparisons are made for default eH parameters, parameters calibrated to provide the best match for SrTiO_3_, and parameters calibrated to provide the best compromise between SrTiO_3_ and SrO.

Density functional	Type of eH parameters	RMSD for *n* = 1, without Sr *d* (eV)	RMSD for *n* = 1, with Sr *d* (eV)	RMSD for *n* = 2, without Sr *d* (eV)	RMSD for *n* = 2, with Sr *d* (eV)	RMSD for *n* = 3, without Sr *d* (eV)	RMSD for *n* = 3, with Sr *d* (eV)
LDA	Default	0.815	0.854	0.775	0.804	0.766	0.790
	SrTiO_3_	0.215	0.225	0.137	0.145	0.108	0.103
	SrTiO_3_/SrO	0.204	0.193	0.155	0.141	0.130	0.125
PBE	Default	0.796	0.788	0.758	0.742	0.750	0.727
	SrTiO_3_	0.192	0.203	0.121	0.130	0.093	0.092
	SrTiO_3_/SrO	0.188	0.194	0.152	0.148	0.134	0.137

**Figure 5:**
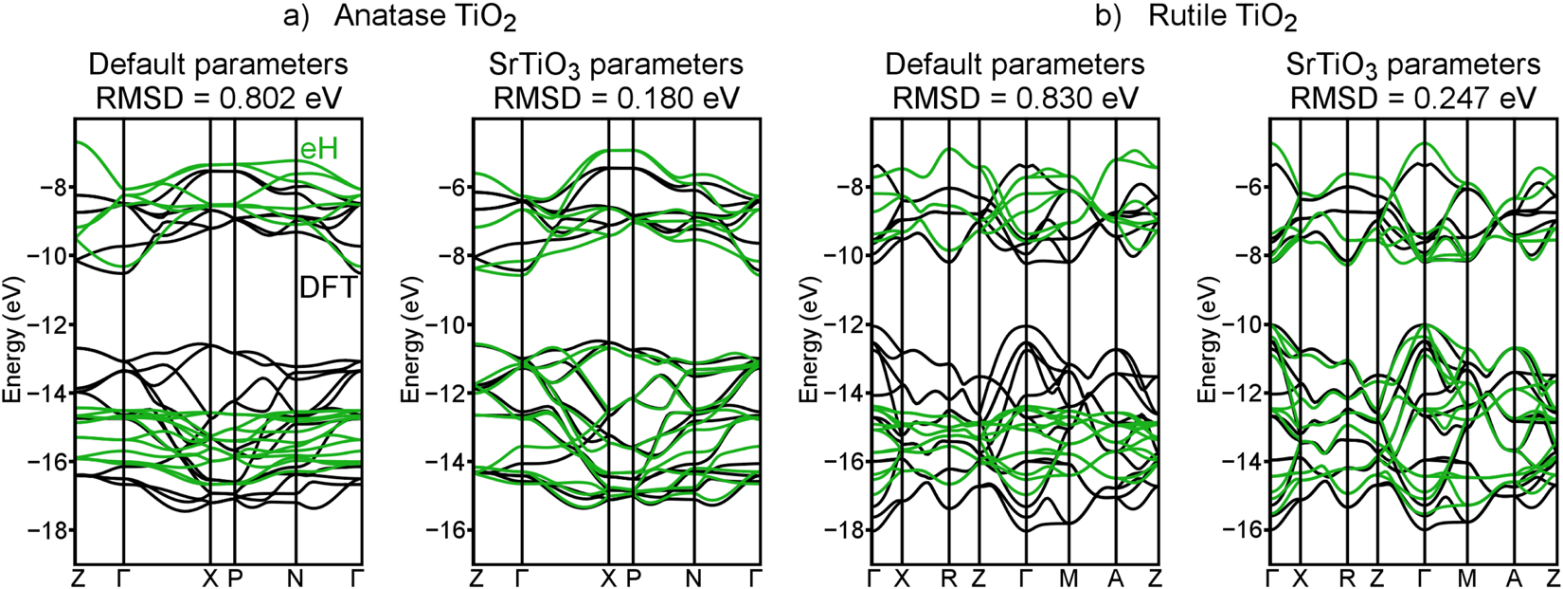
Comparisons of the DFT-LDA (black) and eH (green) band structures of TiO_2_ in the (**a**) anatase and (**b**) rutile structures. Default eH parameters are shown on the left, and parameters calibrated for SrTiO_3_ are shown on the right.

Finally, we move to two phases that are less directly related to those used in calibrating our eH parameters: the anatase (Fig. 1d) and rutile (Fig. 1e) phases of TiO_2_. While their structural similarities to SrTiO_3_ and SrO are less obvious, both TiO_2_ structures (like SrTiO_3_ and the Ruddlesden-Popper phases) have Ti ^4+^ cations that are approximately octahedrally coordinated by O ^2−^ anions. In Fig. 5, band structures of anatase and rutile TiO_2_ computed using the eH method are compared to those computed using DFT-LDA. Once again, default eH parameters (Fig. 5, left) yield poor matches to DFT, with RMSDs of more than 0.8 eV and significant qualitative differences for both anatase and rutile. Because TiO_2_ possesses more elemental and structural similarity to SrTiO_3_ than to SrO, we posit that it is preferable to apply the eH parameters calibrated using only SrTiO_3_. With these calibrated parameters (which included Sr 4*d* orbitals in the SrTiO_3_ calibration process), the RMSDs of both TiO_2_ phases fall below 0.25 eV, visibly improving the match between DFT and eH in terms of band shapes and energies (Fig. 5, right).

Table 4 summarizes the extent to which various eH parameters match DFT calculations of anatase and rutile TiO_2_ using the LDA and PBE functionals. Again, there is no significant difference in results for the two functionals, and no consistent benefit to including Sr 4*d* orbitals in the calibration process. Due to the greater similarity of TiO_2_ to SrTiO_3_ than to SrO, our results confirm that parameters calibrated using only SrTiO_3_ capture the TiO_2_ band structures more effectively than those calibrated by simultaneously considering SrTiO_3_ and SrO. The eH parameter sets corresponding to the results in Table 4 are given in the Supplementary Information.

**Table 4:** Root-mean-squared deviations (RMSDs) of eH band structures of TiO_2_ (in both its anatase and rutile structures) from their DFT counterparts. Comparisons are made for default eH parameters, parameters calibrated to provide the best match for SrTiO_3_, and parameters calibrated to provide the best compromise between SrTiO_3_ and SrO.

Density functional	Type of eH parameters	RMSD for anatase, without Sr *d* (eV)	RMSD for anatase, with Sr *d* (eV)	RMSD for rutile, without Sr *d* (eV)	RMSD for rutile, with Sr *d* (eV)
LDA	Default	0.802	—	0.830	—
	SrTiO_3_	0.172	0.180	0.230	0.247
	SrTiO_3_/SrO	0.206	0.292	0.283	0.277
PBE	Default	0.802	—	0.815	—
	SrTiO_3_	0.181	0.162	0.215	0.233
	SrTiO_3_/SrO	0.181	0.285	0.267	0.287

### Screening of Electronic Band Gaps

We have shown that, when eH input parameters are calibrated, the individual band energies within DFT and eH band structures match quite closely. This positions the eH method well as a screening tool for properties that depend on density of states. As a further demonstration of the screening value of calibrated eH calculations, we turn our attention to band gaps. Because a material’s band gap is often viewed as a proxy for its ability to absorb solar energy, a method that screens band gaps quickly and accurately can be extremely powerful. In Table 5, we assess the ability of our approach to accurately predict band gaps. In this table, computed band gaps are compared for DFT-LDA calculations, eH calculations with default parameters, and eH calculations with parameters calibrated based on the band structure of SrTiO_3_. We focus on parameters calibrated based only on SrTiO_3_ (and omit SrO from the table altogether) because our calibrations of SrO compared only the valence bands, and are therefore not informative regarding the band gap. For brevity, Table 5 shows only the comparisons of DFT-LDA to eH with Sr 4*d* orbitals included in the basis set. More complete results, which support the same qualitative conclusions, are given in the Supplementary Information.

**Table 5:** Comparisons of band gaps computed using DFT-LDA, eH with default parameters (including Sr 4*d* orbitals), and eH with parameters calibrated based on the band structure of SrTiO_3_.

Compound	Method	Band gap, *E*_*g*_ (eV)	*E*_*g*_(eH)−*E*_*g*_(DFT) (eV)
SrTiO_3_	DFT-LDA	1.81	—
	eH, default	3.26	1.46
	eH, calibrated	1.88	0.077
Sr_2_ TiO_4_ (*n* = 1 RP phase)	DFT-LDA	1.97	—
	eH, default	2.46	0.486
	eH, calibrated	2.02	0.046
Sr_3_ Ti_2_ O_7_ (*n* = 2 RP phase)	DFT-LDA	1.89	—
	eH, default	2.65	0.761
	eH, calibrated	1.96	0.074
Sr_4_ Ti_3_ O_10_ (*n* = 3 RP phase)	DFT-LDA	1.82	—
	eH, default	2.68	0.863
	eH, calibrated	1.93	0.109
Anatase TiO_2_	DFT-LDA	2.10	—
	eH, default	4.12	2.02
	eH, calibrated	2.00	−0.101
Rutile TiO_2_	DFT-LDA	1.81	—
	eH, default	4.54	2.73
	eH, calibrated	1.75	−0.061

There are several trends worth noting in Table 5. First, the band gaps computed using default eH parameters are not at all predictive of DFT-computed gaps. For the Sr-Ti-O compounds we study in this paper, default eH gaps are significantly larger than DFT gaps, sometimes by more than 2 eV. Second, the calibrated eH band gaps shown in Table 5 match DFT gaps within approximately 0.1 eV. Given the huge computational savings, eH band gap calculations of this quality can be a useful quantitative screening tool. Third, the calibrated eH calculations (but not the default eH calculations) capture important chemical trends within the compounds studied. For example, calibrated eH calculations correctly predict that band gaps become narrower as *n* increases in the Ruddlesden-Popper series, and that rutile has a band gap significantly smaller than that of anatase.

## Conclusions

One can take away several lessons from this work. First, sets of appropriately calibrated, transferable eH parameters can produce band structures whose bands quantitatively match DFT calculations within about two tenths of an eV, at a tiny fraction of the computational cost of DFT. This is a close enough match to suggest that the eH method has potential use as a tool to approximately screen electronic properties. Second, one must use caution when choosing the compounds with which to calibrate eH parameters. As our work has shown, eH parameters are most transferable when the compounds of interest have as much structural similarity as possible to those used in the calibration process. Given the simplicity, approximations, and relatively small number of parameters used in the eH method, one could hardly expect a universal set of eH parameters to capture the quantum mechanical complexities of every possible chemical environment of every element. Finally, in addition to the extreme computational savings in using the eH method for complex superstructures and nanostructures, we note that unlike DFT and *ab initio* wavefunction-based methods (and machine learning approaches), the chemical simplicity of the eH method easily lends itself to qualitative, transparent orbital-based understanding.

## Electronic supplementary material

Supplementary information
