# Supplementary material for: Calibrating the Extended Hückel Method to Quantitatively Screen the Electronic Properties of Materials
Source: Sci Rep. 2018 Jul 12;8:10530. doi: 10.1038/s41598-018-28864-2 (PMC6043563; doi:10.1038/s41598-018-28864-2)
Supplement: Supplementary file 1 — Supplementary information [file 41598_2018_28864_MOESM1_ESM.pdf]

Supplementary Information for "Calibrating the Extended Hückel Method to Quantitatively Screen the Electronic Properties of Materials"

Linda P. Grabill and Robert F. Berger*

Department of Chemistry, Western Washington University, Bellingham, WA

E-mail: Robert.Berger@wwu.edu

*To whom correspondence should be addressed

Extended Hückel methodology

Because this work focuses on developing physically meaningful and transferable sets of eH input parameters, it is useful to briefly acquaint ourselves with the eH methodology and the associated input parameters. A full treatment is given in the original work of Hoffmann.[1]

In the eH method, the linear combination of atomic orbital (LCAO) secular equations for a system with n basis orbitals can be written as:

$$\begin{pmatrix} \int \phi_1 H \phi_1 \mathrm{d}\tau & \dots & \int \phi_1 H \phi_n \mathrm{d}\tau \\ \vdots & \ddots & \vdots \\ \int \phi_n H \phi_1 \mathrm{d}\tau & \dots & \int \phi_n H \phi_n \mathrm{d}\tau \end{pmatrix} \begin{pmatrix} c_1 \\ \vdots \\ c_n \end{pmatrix} = E \begin{pmatrix} \int \phi_1 \phi_1 \mathrm{d}\tau & \dots & \int \phi_1 \phi_n \mathrm{d}\tau \\ \vdots & \ddots & \vdots \\ \int \phi_n \phi_1 \mathrm{d}\tau & \dots & \int \phi_n \phi_n \mathrm{d}\tau \end{pmatrix} \begin{pmatrix} c_1 \\ \vdots \\ c_n \end{pmatrix}$$

or more concisely, $\boldsymbol{H}\vec{c} = E\boldsymbol{S}\vec{c}$. This system of equations is solved for the crystal orbital energies (E) and the atomic orbital coefficients (c_j) in each crystal orbital. The unique character of the eH method lies in the manner in which the Hamiltonian matrix ($\boldsymbol{H}$) and overlap matrix ($\boldsymbol{S}$) are populated. The diagonal elements of the overlap matrix (S_{ii}) are set to 1, as the basis atomic orbitals are normalized. The off-diagonal elements (S_{ij}) are populated with numerically computed overlap integrals, $\int \phi_i \phi_j \mathrm{d}\tau$. The diagonal elements of the Hamiltonian matrix (H_{ii}) are input parameters characteristic of the type of atomic orbital in question. Traditionally, these values are taken to be experimental ionization energies. In this work, we allow them to vary as adjustable parameters. The off-diagonal elements (H_{ij}) are often populated using the Wolfsberg-Helmholtz approximation:

$$H_{ij} = KS_{ij}\frac{H_{ii} + H_{jj}}{2}$$

Our calculations use YAeHMOP's modified form of H_{ij}, which reduces the effect of counter-intuitive orbital mixing.[2] While K is traditionally taken to be 1.75, we allow K to vary as an adjustable parameter.

The remaining input parameters in an eH calculation characterize the atomic orbital basis functions. Only the valence atomic orbitals of each atom are treated explicitly, and they are represented by Slater-type orbitals. Valence s and p orbitals take the form of single-ζ functions:

$$\psi^{n,l,m}(r,\theta,\phi) = Nr^{n-1}e^{-\zeta r}Y^{l,m}(\theta,\phi)$$

Where applicable, valence d orbitals take the form of double-ζ functions:

$$\psi^{n,l,m}(r,\theta,\phi) = Nr^{n-1}(c_1e^{-\zeta_1 r} + c_2e^{-\zeta_2 r})Y^{l,m}(\theta,\phi)$$

Therefore, each element's valence s orbital has (in addition to its "energy" H_{ii}) one parameter, ζ_s, which governs its spatial extent. Each element's set of valence p orbitals also has (in addition to H_{ii}) an associated ζ_p. Because d orbitals are represented by double-ζ functions, each element's set of valence d orbitals has (in addition to H_{ii}) an associated ζ_1, ζ_2, and c_2/c_1, the ratio of their coefficients. The coefficients themselves are then fixed by the fact that the basis orbitals are normalized.

DFT-optimized crystal structures

The first 16 tables of the Supporting Information provide the DFT-optimized crystal structures (in VASP POSCAR format) of $SrTiO_3$, SrO, the $n = 1 - 3$ members of the Sr–Ti–O Ruddlesden-Popper series, and anatase and rutile TiO_2.

Table S1: VASP structure file for $SrTiO_3$ optimized using DFT-LDA.

$SrTiO_3$ DFT-LDA			
1.00000000000000			
	3.8606564586281324	0.0000000000000000	0.0000000000000000
	0.0000000000000000	3.8606564586281324	0.0000000000000000
	0.0000000000000000	0.0000000000000000	3.8606564586281324
Sr Ti O			
1 1 3			
Direct			
	0.0000000000000000	0.0000000000000000	0.0000000000000000
	0.5000000000000000	0.5000000000000000	0.5000000000000000
	0.5000000000000000	0.5000000000000000	0.0000000000000000
	0.0000000000000000	0.5000000000000000	0.5000000000000000
	0.5000000000000000	0.0000000000000000	0.5000000000000000

Table S2: VASP structure file for $SrTiO_3$ optimized using DFT-PBE.

$SrTiO_3$ DFT-PBE			
1.00000000000000			
	3.9404294239672404	0.0000000000000000	0.0000000000000000
	0.0000000000000000	3.9404294239672404	0.0000000000000000
	0.0000000000000000	0.0000000000000000	3.9404294239672404
Sr Ti O			
1 1 3			
Direct			
	0.0000000000000000	0.0000000000000000	0.0000000000000000
	0.5000000000000000	0.5000000000000000	0.5000000000000000
	0.5000000000000000	0.5000000000000000	0.0000000000000000
	0.0000000000000000	0.5000000000000000	0.5000000000000000
	0.5000000000000000	0.0000000000000000	0.5000000000000000

Table S3: VASP structure file for $SrTiO_3$ optimized using DFT-HSE06.

$SrTiO_3$ DFT-HSE06
1.00000000000000

3.8982145621754585	0.0000000000000000	0.0000000000000000
0.0000000000000000	3.8982145621754585	0.0000000000000000
0.0000000000000000	0.0000000000000000	3.8982145621754585

Sr Ti O
1 1 3
Direct

0.0000000000000000	0.0000000000000000	0.0000000000000000
0.5000000000000000	0.5000000000000000	0.5000000000000000
0.5000000000000000	0.5000000000000000	0.0000000000000000
0.0000000000000000	0.5000000000000000	0.5000000000000000
0.5000000000000000	0.0000000000000000	0.5000000000000000

Table S4: VASP structure file for SrO optimized using DFT-LDA.

SrO DFT-LDA
1.00000000000000

2.5389361449567316	2.5389361449567316	0.0000000000000000
0.0000000000000000	2.5389361449567316	2.5389361449567316
2.5389361449567316	0.0000000000000000	2.5389361449567316

Sr O
1 1
Direct

0.0000000000000000	0.0000000000000000	0.0000000000000000
0.5000000000000000	0.5000000000000000	0.5000000000000000

Table S5: VASP structure file for SrO optimized using DFT-PBE.

SrO DFT-PBE
1.00000000000000

2.6018495867395042	2.6018495867395042	0.0000000000000000
0.0000000000000000	2.6018495867395042	2.6018495867395042
2.6018495867395042	0.0000000000000000	2.6018495867395042

Sr O
1 1
Direct

0.0000000000000000	0.0000000000000000	0.0000000000000000
0.5000000000000000	0.5000000000000000	0.5000000000000000

Table S6: VASP structure file for SrO optimized using DFT-HSE06.

```
SrO DFT-HSE06
1.00000000000000
    2.5816536337761562    2.5816536337761562    0.0000000000000000
    0.0000000000000000    2.5816536337761562    2.5816536337761562
    2.5816536337761562    0.0000000000000000    2.5816536337761562
Sr   O
1    1
Direct
    0.0000000000000000    0.0000000000000000    0.0000000000000000
    0.5000000000000000    0.5000000000000000    0.5000000000000000
```

Table S7: VASP structure file for Sr_2TiO_4 (the $n = 1$ Ruddlesden-Popper phase) optimized using DFT-LDA.

```
Sr2TiO4 DFT-LDA
1.00000000000000
    3.8377975869403982    0.0000000000000000    0.0000000000000000
    0.0000000000000000    3.8377975869403982    0.0000000000000000
    1.9188987934701991    1.9188987934701991    6.1982053881684935
Sr   Ti   O
2    1    4
Direct
    0.8548051157629217    0.8548051157629217    0.2903897684741494
    0.1451948842370783    0.1451948842370783    0.7096102315258506
    0.5000000000000000    0.5000000000000000    0.0000000000000000
    0.5000000000000000    0.0000000000000000    0.0000000000000000
    0.0000000000000000    0.5000000000000000    0.0000000000000000
    0.3411629135245349    0.3411629135245349    0.3176741729509232
    0.6588370864754651    0.6588370864754651    0.6823258270490697
```

Table S8: VASP structure file for Sr_2TiO_4 (the $n = 1$ Ruddlesden-Popper phase) optimized using DFT-PBE.

```
Sr2TiO4 DFT-PBE
1.00000000000000
    3.9234142446128808    0.0000000000000000    0.0000000000000000
    0.0000000000000000    3.9234142446128808    0.0000000000000000
    1.9617071223064404    1.9617071223064404    6.3389037546532077
Sr   Ti   O
2    1    4
Direct
    0.8546812161411808    0.8546812161411808    0.2906375677176456
    0.1453187838588192    0.1453187838588192    0.7093624322823544
    0.5000000000000000    0.5000000000000000    0.0000000000000000
    0.5000000000000000    0.0000000000000000    0.0000000000000000
    0.0000000000000000    0.5000000000000000    0.0000000000000000
    0.3419395038789830    0.3419395038789830    0.3161209922420340
    0.6580604961210170    0.6580604961210170    0.6838790077579660
```

Table S9: VASP structure file for $Sr_3Ti_2O_7$ (the $n = 2$ Ruddlesden-Popper phase) optimized using DFT-LDA.

```
Sr3Ti2O7 DFT-LDA
1.00000000000000
    3.8532417154806087    0.0000000000000000    0.0000000000000000
    0.0000000000000000    3.8532417154806087    0.0000000000000000
    1.9266208577403043    1.9266208577403043    10.0297100869265652
Sr   Ti   O
3    2    7
Direct
    0.0843044910782922    0.0843044910782922    0.8313910178434156
    0.9000000000000000    0.9000000000000000    0.2000000000000000
    0.7156955089217121    0.7156955089217121    0.5686089821565830
    0.4976778026654500    0.4976778026654500    0.0046443946690928
    0.3023221973345471    0.3023221973345471    0.3953556053309057
    0.5942853454234367    0.5942853454234367    0.8114293091531195
    0.4000000000000000    0.4000000000000000    0.2000000000000000
    0.2057146545765676    0.2057146545765676    0.5885706908468791
    0.4960604173323944    0.9960604173323944    0.0078791653352184
    0.9960604173323944    0.4960604173323944    0.0078791653352184
    0.3039395826676028    0.8039395826676028    0.3921208346647802
    0.8039395826676028    0.3039395826676028    0.3921208346647802
```

Table S10: VASP structure file for $Sr_3Ti_2O_7$ (the $n = 2$ Ruddlesden-Popper phase) optimized using DFT-PBE.

$Sr_3Ti_2O_7$ DFT-PBE
1.00000000000000

3.9359925871004604	0.0000000000000000	0.0000000000000000
0.0000000000000000	3.9359925871004604	0.0000000000000000
1.9679962935502302	1.9679962935502302	10.2457608261301321

Sr Ti O
3 2 7
Direct

0.0842924295072436	0.0842924295072436	0.8314151409855057
0.9000000000000000	0.9000000000000000	0.2000000000000000
0.7157075704927607	0.7157075704927607	0.5685848590144928
0.4976928105564724	0.4976928105564724	0.0046143788870481
0.3023071894435247	0.3023071894435247	0.3953856211129505
0.5936825725828498	0.5936825725828498	0.8126348548343003
0.4000000000000000	0.4000000000000000	0.2000000000000000
0.2063174274171544	0.2063174274171544	0.5873651451656983
0.4963226323486154	0.9963226323486154	0.0073547353027763
0.9963226323486154	0.4963226323486154	0.0073547353027763
0.3036773676513818	0.8036773676513818	0.3926452646972223
0.8036773676513818	0.3036773676513818	0.3926452646972223

Table S11: VASP structure file for $Sr_4Ti_3O_{10}$ (the $n = 3$ Ruddlesden-Popper phase) optimized using DFT-LDA.

```
Sr4Ti3O10 DFT-LDA
1.00000000000000
    3.8558959036977170    0.0000000000000000    0.0000000000000000
    0.0000000000000000    3.8558959036977170    0.0000000000000000
    1.9279479518488585    1.9279479518488585   13.8838106152119209
Sr  Ti  O
4   3   10
Direct
    0.0595476100252270    0.0595476100252270    0.8809047799495460
    0.9257518175906725    0.9257518175906725    0.1484963648186621
    0.7885338966950428    0.7885338966950428    0.4229322066099144
    0.6547381042604883    0.6547381042604883    0.6905237914790234
    0.4974700876703508    0.4974700876703508    0.0050598246592983
    0.3571428571428541    0.3571428571428541    0.2857142857142847
    0.2168156266153645    0.2168156266153645    0.5663687467692711
    0.5670135802643159    0.5670135802643159    0.8659728394713611
    0.4267634713382407    0.4267634713382407    0.1464730573235116
    0.2875222429474746    0.2875222429474746    0.4249555141050649
    0.1472721340213994    0.1472721340213994    0.7054557319572083
    0.4961134311950843    0.9961134311950843    0.0077731376098313
    0.9961134311950843    0.4961134311950843    0.0077731376098313
    0.3571428571428541    0.8571428571428541    0.2857142857142847
    0.8571428571428541    0.3571428571428541    0.2857142857142847
    0.2181722830906310    0.7181722830906310    0.5636554338187381
    0.7181722830906310    0.2181722830906310    0.5636554338187381
```

Table S12: VASP structure file for $Sr_4Ti_3O_{10}$ (the $n = 3$ Ruddlesden-Popper phase) optimized using DFT-PBE.

$Sr_4Ti_3O_{10}$ DFT-PBE
1.00000000000000

3.9391010376671187	0.0000000000000000	0.0000000000000000
0.0000000000000000	3.9391010376671187	0.0000000000000000
1.9695505188335594	1.9695505188335594	14.1820236062497678

Sr Ti O
4 3 10
Direct

0.0595614339357482	0.0595614339357482	0.8808771321285036
0.9256549891855741	0.9256549891855741	0.1486900216288589
0.7886307251001412	0.7886307251001412	0.4227385497997176
0.6547242803499671	0.6547242803499671	0.6905514393000658
0.4974850864271545	0.4974850864271545	0.0050298271456839
0.3571428571428541	0.3571428571428541	0.2857142857142847
0.2168006278585608	0.2168006278585608	0.5663987442828855
0.5666204177626213	0.5666204177626213	0.8667591644747432
0.4266678575096279	0.4266678575096279	0.1466642849807371
0.2876178567760874	0.2876178567760874	0.4247642864478394
0.1476652965230940	0.1476652965230940	0.7046694069538262
0.4962725561479431	0.9962725561479431	0.0074548877041138
0.9962725561479431	0.4962725561479431	0.0074548877041138
0.3571428571428541	0.8571428571428541	0.2857142857142847
0.8571428571428541	0.3571428571428541	0.2857142857142847
0.2180131581377722	0.7180131581377722	0.5639736837244556
0.7180131581377722	0.2180131581377722	0.5639736837244556

Table S13: VASP structure file for anatase TiO_2 optimized using DFT-LDA.

Anatase TiO_2 DFT-LDA
1.00000000000000

3.7461982332075938	0.0000000000000000	0.0000000000000000
0.0000000000000000	3.7461982332075938	0.0000000000000000
1.8730991166037969	1.8730991166037969	4.7353991945400606

Ti O
2 4
Direct

0.0000000000000000	0.0000000000000000	0.0000000000000000
0.7500000000000000	0.2500000000000000	0.5000000000000000
0.7920287602144640	0.7920287602144640	0.4159424795710649
0.5420287601801519	0.0420287601534568	0.9159424796396891
0.9579712397512168	0.4579712397245288	0.0840575204975664
0.2079712397957962	0.2079712397957962	0.5840575203550316

Table S14: VASP structure file for anatase TiO_2 optimized using DFT-PBE.

Anatase TiO_2 DFT-PBE
1.00000000000000

3.8146223043692911	0.0000000000000000	0.0000000000000000
0.0000000000000000	3.8146223043692911	0.0000000000000000
1.9073111521846455	1.9073111521846455	4.8104318525633101

Ti O
2 4
Direct

0.0000000000000000	0.0000000000000000	0.0000000000000000
0.7500000000000000	0.2500000000000000	0.5000000000000000
0.7922311706481793	0.7922311706481793	0.4155376587036272
0.5422311706138672	0.0422311705871721	0.9155376587722515
0.9577688293175015	0.4577688292908135	0.0844623413650041
0.2077688293620810	0.2077688293620810	0.5844623412224692

Table S15: VASP structure file for rutile TiO_2 optimized using DFT-LDA.

Rutile TiO_2 DFT-LDA
1.00000000000000

4.5531729170813149	0.0000000000000000	0.0000000000000000
0.0000000000000000	4.5531729170813149	0.0000000000000000
0.0000000000000000	0.0000000000000000	2.9228290700207573

Ti O
2 4
Direct

0.0000000000000000	0.0000000000000000	0.0000000000000000
0.5000000000000000	0.5000000000000000	0.5000000000000000
0.3038355782439766	0.3038355782439766	0.0000000000000000
0.6961644217560234	0.6961644217560234	0.0000000000000000
0.1961644217560234	0.8038355782439766	0.5000000000000000
0.8038355782439766	0.1961644217560234	0.5000000000000000

Table S16: VASP structure file for rutile TiO_2 optimized using DFT-PBE.

Rutile TiO_2 DFT-PBE
1.00000000000000

4.6458092581807273	0.0000000000000000	0.0000000000000000
0.0000000000000000	4.6458092581807273	0.0000000000000000
0.0000000000000000	0.0000000000000000	2.9701777676336394

Ti O
2 4
Direct

0.0000000000000000	0.0000000000000000	0.0000000000000000
0.5000000000000000	0.5000000000000000	0.5000000000000000
0.3050472625408034	0.3050472625408034	0.0000000000000000
0.6949527374591966	0.6949527374591966	0.0000000000000000
0.1949527374591966	0.8050472625408034	0.5000000000000000
0.8050472625408034	0.1949527374591966	0.5000000000000000

Default and calibrated eH parameters

The next 14 tables of the Supporting Information provide sets of default and calibrated YAeHMOP eH parameters.

Table S17: Default YAeHMOP eH parameters of Sr (without $4d$), Ti, and O.

Orbital	H_{ii}	ζ_1	ζ_2	c_2/c_1
Sr $5s$	−6.620000	1.214000		
Sr $5p$	−3.920000	1.214000		
Ti $4s$	−8.970000	1.075000		
Ti $4p$	−5.440000	1.075000		
Ti $3d$	−10.810000	4.550000	1.400000	1.863766
O $2s$	−32.300000	2.275000		
O $2p$	−14.800000	2.275000		
$K = 1.750000$				

Table S18: Calibrated eH parameters of Sr (without $4d$), Ti, and O, based on the DFT-LDA band structure of $SrTiO_3$.

Orbital	H_{ii}	ζ_1	ζ_2	c_2/c_1
Sr $5s$	−4.686617	2.438235		
Sr $5p$	−0.480387	1.777769		
Ti $4s$	−2.302619	1.572930		
Ti $4p$	−1.924475	5.093655		
Ti $3d$	−8.497439	3.796660	2.040961	1.811097
O $2s$	−24.036623	3.537245		
O $2p$	−12.008070	1.998022		
$K = 2.690660$				

Table S19: Calibrated eH parameters of Sr (without $4d$), Ti, and O, based on the DFT-PBE band structure of $SrTiO_3$.

Orbital	H_{ii}	ζ_1	ζ_2	c_2/c_1
Sr $5s$	-3.845618	2.292611		
Sr $5p$	-0.482132	1.737511		
Ti $4s$	-2.220216	1.664349		
Ti $4p$	-1.569297	3.630481		
Ti $3d$	-8.020648	6.405972	2.108367	2.080233
O $2s$	-18.943833	3.726857		
O $2p$	-11.366348	1.972051		
$K = 2.751933$				

Table S20: Calibrated eH parameters of Sr (without $4d$), Ti, and O, based on the DFT-HSE06 band structure of $SrTiO_3$.

Orbital	H_{ii}	ζ_1	ζ_2	c_2/c_1
Sr $5s$	-3.995970	2.459402		
Sr $5p$	-0.388849	1.878675		
Ti $4s$	-2.014861	1.618307		
Ti $4p$	-3.823866	4.475218		
Ti $3d$	-7.649746	3.276826	1.999509	1.417818
O $2s$	-26.351528	3.815349		
O $2p$	-12.780646	2.036702		
$K = 3.043504$				

Table S21: Calibrated eH parameters of Sr (without $4d$), Ti, and O, based on the DFT-LDA band structures of $SrTiO_3$ and SrO.

Orbital	H_{ii}	ζ_1	ζ_2	c_2/c_1
Sr $5s$	-6.355384	2.757655		
Sr $5p$	-0.214851	0.938124		
Ti $4s$	-4.760989	2.818806		
Ti $4p$	-1.481318	1.792491		
Ti $3d$	-8.709574	6.049505	1.908037	1.074333
O $2s$	-33.502252	4.401109		
O $2p$	-11.581395	2.146488		
$K = 2.955321$				

Table S22: Calibrated eH parameters of Sr (without $4d$), Ti, and O, based on the DFT-PBE band structures of $SrTiO_3$ and SrO.

Orbital	H_{ii}	ζ_1	ζ_2	c_2/c_1
Sr $5s$	−6.263232	2.780532		
Sr $5p$	−0.184912	0.915282		
Ti $4s$	−3.307911	2.744368		
Ti $4p$	−1.456664	1.670962		
Ti $3d$	−8.294601	7.566714	1.908176	0.946004
O $2s$	−31.975554	4.713500		
O $2p$	−10.838759	2.213470		
		$K = 3.319161$		

Table S23: Calibrated eH parameters of Sr (without $4d$), Ti, and O, based on the DFT-HSE06 band structures of $SrTiO_3$ and SrO.

Orbital	H_{ii}	ζ_1	ζ_2	c_2/c_1
Sr $5s$	−4.712053	1.281403		
Sr $5p$	−0.612409	0.693560		
Ti $4s$	−5.513911	1.975343		
Ti $4p$	−5.279121	2.757285		
Ti $3d$	−8.301133	4.518567	1.721606	1.984061
O $2s$	−31.114254	5.101600		
O $2p$	−12.116840	2.245921		
		$K = 2.874517$		

Table S24: Default YAeHMOP eH parameters of Sr (including $4d$), Ti, and O.

Orbital	H_{ii}	ζ_1	ζ_2	c_2/c_1
Sr $5s$	−6.620000	1.214000		
Sr $5p$	−3.920000	1.214000		
Sr $4d$	−11.180000	3.835000	1.505000	0.928986
Ti $4s$	−8.970000	1.075000		
Ti $4p$	−5.440000	1.075000		
Ti $3d$	−10.810000	4.550000	1.400000	1.863766
O $2s$	−32.300000	2.275000		
O $2p$	−14.800000	2.275000		
		$K = 1.750000$		

Table S25: Calibrated eH parameters of Sr (including $4d$), Ti, and O, based on the DFT-LDA band structure of $SrTiO_3$.

Orbital	H_{ii}	ζ_1	ζ_2	c_2/c_1
Sr $5s$	−3.118926	1.539482		
Sr $5p$	−0.935945	1.816254		
Sr $4d$	−3.877334	8.939981	1.335216	0.885669
Ti $4s$	−2.914726	1.764828		
Ti $4p$	−0.764167	1.994679		
Ti $3d$	−8.401423	2.488894	2.156772	1.434110
O $2s$	−34.582331	3.558012		
O $2p$	−11.679064	2.023445		
$K = 2.713291$				

Table S26: Calibrated eH parameters of Sr (including $4d$), Ti, and O, based on the DFT-PBE band structure of $SrTiO_3$.

Orbital	H_{ii}	ζ_1	ζ_2	c_2/c_1
Sr $5s$	−2.892598	1.560894		
Sr $5p$	−2.861981	2.911556		
Sr $4d$	−3.700272	7.397931	1.232217	0.744109
Ti $4s$	−3.070164	1.926431		
Ti $4p$	−0.959629	1.934534		
Ti $3d$	−7.965213	2.392855	2.134900	0.993078
O $2s$	−37.197996	3.674620		
O $2p$	−11.105659	1.987163		
$K = 2.709863$				

Table S27: Calibrated eH parameters of Sr (including $4d$), Ti, and O, based on the DFT-HSE06 band structure of $SrTiO_3$.

Orbital	H_{ii}	ζ_1	ζ_2	c_2/c_1
Sr $5s$	−2.445929	1.666025		
Sr $5p$	−0.835954	1.832617		
Sr $4d$	−3.301940	7.102273	1.230252	0.735259
Ti $4s$	−2.651052	1.641559		
Ti $4p$	−1.072051	2.435853		
Ti $3d$	−7.632240	3.232547	2.138056	4.224683
O $2s$	−35.151580	4.066375		
O $2p$	−12.456529	2.066211		
$K = 3.058686$				

Table S28: Calibrated eH parameters of Sr (including $4d$), Ti, and O, based on the DFT-LDA band structures of $SrTiO_3$ and SrO.

Orbital	H_{ii}	ζ_1	ζ_2	c_2/c_1
Sr $5s$	−5.539883	2.297406		
Sr $5p$	1.432011	6.163659		
Sr $4d$	−6.185784	13.589997	1.710941	0.531727
Ti $4s$	−5.013404	2.520183		
Ti $4p$	−3.282152	1.164429		
Ti $3d$	−8.870410	5.746328	1.870382	1.872296
O $2s$	−33.641935	4.926647		
O $2p$	−11.112597	2.277723		
		$K = 2.981200$		

Table S29: Calibrated eH parameters of Sr (including $4d$), Ti, and O, based on the DFT-PBE band structures of $SrTiO_3$ and SrO.

Orbital	H_{ii}	ζ_1	ζ_2	c_2/c_1
Sr $5s$	−5.720580	2.321560		
Sr $5p$	−0.002117	1.932703		
Sr $4d$	−5.993195	17.288292	1.683764	0.446839
Ti $4s$	−4.712012	2.173539		
Ti $4p$	−2.980960	1.237703		
Ti $3d$	−8.391410	11.713104	1.863242	1.889867
O $2s$	−28.004058	4.972875		
O $2p$	−10.508832	2.262507		
		$K = 2.916468$		

Table S30: Calibrated eH parameters of Sr (including $4d$), Ti, and O, based on the DFT-HSE06 band structures of $SrTiO_3$ and SrO.

Orbital	H_{ii}	ζ_1	ζ_2	c_2/c_1
Sr $5s$	−4.900789	2.141045		
Sr $5p$	1.388760	5.903623		
Sr $4d$	−5.248505	13.313522	1.605326	0.493843
Ti $4s$	−5.749662	2.414076		
Ti $4p$	−3.301041	1.245504		
Ti $3d$	−8.204550	5.608176	1.717191	2.089238
O $2s$	−34.446085	5.000109		
O $2p$	−11.767180	2.463041		
		$K = 3.344787$		

Band gap comparisons

The last two tables of the Supporting Information compare the band gaps of various compounds computed using DFT and default and calibrated eH parameters.

Table S31: Comparisons of band gaps computed using DFT-LDA, eH with default parameters (without and with Sr $4d$ orbitals), and eH with parameters calibrated based on the band structure of $SrTiO_3$.

Compound	Method	Band gap, E_g (eV)	E_g(eH)$-E_g$(DFT) (eV)
$SrTiO_3$	DFT-LDA	1.81	—
	eH, default (without Sr $4d$)	4.76	2.95
	eH, calibrated (without Sr $4d$)	2.14	0.330
	eH, default (with Sr $4d$)	3.26	1.46
	eH, calibrated (with Sr $4d$)	1.88	0.077
Sr_2TiO_4	DFT-LDA	1.97	—
($n = 1$	eH, default (without Sr $4d$)	4.84	2.87
RP phase)	eH, calibrated (without Sr $4d$)	2.08	0.110
	eH, default (with Sr $4d$)	2.46	0.486
	eH, calibrated (with Sr $4d$)	2.02	0.046
$Sr_3Ti_2O_7$	DFT-LDA	1.89	—
($n = 2$	eH, default (without Sr $4d$)	4.80	2.91
RP phase)	eH, calibrated (without Sr $4d$)	2.12	0.229
	eH, default (with Sr $4d$)	2.65	0.761
	eH, calibrated (with Sr $4d$)	1.96	0.074
$Sr_4Ti_3O_{10}$	DFT-LDA	1.82	—
($n = 3$	eH, default (without Sr $4d$)	4.79	2.97
RP phase)	eH, calibrated (without Sr $4d$)	2.12	0.305
	eH, default (with Sr $4d$)	2.68	0.863
	eH, calibrated (with Sr $4d$)	1.93	0.109
Anatase	DFT-LDA	2.10	—
TiO_2	eH, default	4.12	2.02
	eH, calibrated (without Sr $4d$)	1.91	−0.192
	eH, calibrated (with Sr $4d$)	2.00	−0.101
Rutile	DFT-LDA	1.81	—
TiO_2	eH, default	4.54	2.73
	eH, calibrated (without Sr $4d$)	1.76	−0.048
	eH, calibrated (with Sr $4d$)	1.75	−0.061

Table S32: Comparisons of band gaps computed using DFT-PBE, eH with default parameters (without and with Sr $4d$ orbitals), and eH with parameters calibrated based on the band structure of $SrTiO_3$.

Compound	Method	Band gap, E_g (eV)	E_g(eH)$-E_g$(DFT) (eV)
$SrTiO_3$	DFT-PBE	1.80	—
	eH, default (without Sr $4d$)	4.69	2.88
	eH, calibrated (without Sr $4d$)	2.06	0.254
	eH, default (with Sr $4d$)	3.25	1.45
	eH, calibrated (with Sr $4d$)	1.87	0.069
Sr_2TiO_4	DFT-PBE	1.95	—
($n = 1$	eH, default (without Sr $4d$)	4.75	2.80
RP phase)	eH, calibrated (without Sr $4d$)	2.02	0.065
	eH, default (with Sr $4d$)	2.51	0.561
	eH, calibrated (with Sr $4d$)	1.96	0.005
$Sr_3Ti_2O_7$	DFT-PBE	1.88	—
($n = 2$	eH, default (without Sr $4d$)	4.72	2.84
RP phase)	eH, calibrated (without Sr $4d$)	2.05	0.165
	eH, default (with Sr $4d$)	2.70	0.819
	eH, calibrated (with Sr $4d$)	1.93	0.045
$Sr_4Ti_3O_{10}$	DFT-PBE	1.81	—
($n = 3$	eH, default (without Sr $4d$)	4.71	2.90
RP phase)	eH, calibrated (without Sr $4d$)	2.05	0.245
	eH, default (with Sr $4d$)	2.72	0.918
	eH, calibrated (with Sr $4d$)	1.90	0.098
Anatase	DFT-PBE	2.09	—
TiO_2	eH, default	4.08	1.98
	eH, calibrated (without Sr $4d$)	2.06	−0.031
	eH, calibrated (with Sr $4d$)	2.14	0.042
Rutile	DFT-PBE	1.82	—
TiO_2	eH, default	4.46	2.64
	eH, calibrated (without Sr $4d$)	1.72	−0.098
	eH, calibrated (with Sr $4d$)	1.69	−0.128

References

(1) Hoffmann, R. An extended Hückel theory. I. Hydrocarbons. *J. Chem. Phys.* **1963**, *39*, 1397–1412.

(2) Ammeter, J. H.; Bürgi, H.-B.; Thibeault, J. C.; Hoffmann, R. Counterintuitive orbital mixing in semiempirical and *ab initio* molecular orbital calculations. *J. Am. Chem. Soc.* **1978**, *100*, 3686–3692.
